# Fine-Mapping of 18q21.1 Locus Identifies Single Nucleotide Polymorphisms Associated with Nonsyndromic Cleft Lip with or without Cleft Palate

**DOI:** 10.3389/fgene.2016.00088

**Published:** 2016-05-23

**Authors:** Amit K. Mitra, Holly A. F. Stessman, Robert J. Schaefer, Wen Wang, Chad L. Myers, Brian G. Van Ness, Soraya Beiraghi

**Affiliations:** ^1^Department of Genetics, Cell Biology and Development, University of MinnesotaMinneapolis, MN, USA; ^2^Department of Computer Science and Engineering, University of MinnesotaMinneapolis, MN, USA; ^3^Division of Pediatric Dentistry, Department of Developmental and Surgical Science, University of MinnesotaMinneapolis, MN, USA

**Keywords:** clinical genetics, complex traits, exome sequencing, MYO5B, cleft lip

## Abstract

Nonsyndromic cleft lip with or without cleft palate (NSCL/P) is one of the most common congenital birth defects. NSCL/P is a complex multifactorial disease caused by interactions between multiple environmental and genetic factors. However, the causal single nucleotide polymorphism (SNP) signature profile underlying the risk of familial NSCL/P still remains unknown. We previously reported a 5.7-Mb genomic region on chromosome 18q21.1 locus that potentially contributes to autosomal dominant, low-penetrance inheritance of NSCL/P. In the current study, we performed exome sequencing on 12 familial genomes (six affected individuals, two obligate carriers, and four seemingly unaffected individuals) of a six-generation family to identify candidate SNPs associated with NSCL/P risk. Subsequently, targeted bidirectional DNA re-sequencing of polymerase chain reaction (PCR)-amplified high-risk regions of *MYO5B* gene and sequenom iPLEX genotpying of 29 candidate SNPs were performed on a larger set of 33 members of this NSCL/P family (10 affected + 4 obligate carriers + 19 unaffected relatives) to find SNPs significantly associated with NSCL/P trait. SNP vs. NSCL/P association analysis showed the *MYO5B* SNP rs183559995 GA genotype had an odds ratio of 18.09 (95% Confidence Interval = 1.86–176.34; gender-adjusted *P* = 0.0019) compared to the reference GG genotype. Additionally, the following SNPs were also found significantly associated with NSCL/P risk: rs1450425 (*LOXHD1*), rs6507992 (*SKA1*), rs78950893 (*SMAD7*), rs8097060, rs17713847 (*SCARNA17*), rs6507872 (*CTIF*), rs8091995 (*CTIF*), and rs17715416 (*MYO5B*). We could thus identify mutations in several genes as key candidate SNPs associated with the risk of NSCL/P in this large multi-generation family.

## Introduction

Nonsyndromic cleft lip with or without cleft palate (NSCL/P) is one of the most common congenital craniofacial birth defects that accounts for 93–95% cases of Cleft Lip with or without Cleft Palate (CL/P) and represents almost half of facial dysmorphology (Stuppia et al., 2011). NSCL/P consists of isolated, nonspecific malformations of the upper lip and oral cavity and is seen frequently worldwide with average global incidence of 1.7 per 1000 live births and 1 per 700–1000 newborns in the United States each year. Its effect on speech, hearing, appearance, and cognition may cause long-term adverse effects on health and social integration (Mossey et al., 2009). NSCL/P is a multifactorial disease that exhibits a complex etiology due to interactions between multiple genetic and environmental factors. Mutations in several genes have been shown associated with increased risk of NSCL/P in recent years including a causative variant in the promoter region of *IRF6* gene (chromosome 1q32.2) (Rahimov et al., 2012; Leslie et al., 2015). Further, genome-wide linkage analysis and genome-wide association studies (GWAS) have identified and validated association of 13 different genetic loci with the risk of NSCL/P (Leslie et al., 2015). However, the evidences have been largely conflicting and therefore the causal pathogenic variants underlying NSCL/P risk still remains unknown.

Previously, we performed genome-wide linkage analysis on a large multi-generational family of self-reported European origin to identify a 5.7-Mb genomic region on chromosome 18q21.1 that potentially contains a pathogenic, high-risk variant for NSCL/P (Beiraghi et al., 2007). We named this locus as OFC11 or orofacial cleft 11.

In the current study, we performed genetic fine-mapping of the chromosome 18q21.1 region to home in on to the high risk single nucleotide polymorphisms (SNPs) significantly associated with the risk of NSCL/P. Exome sequencing was done on six affected individuals, two obligate carriers, and four unaffected individuals from the NSCL/P family that identified candidate SNPs including multiple highly significant SNPs within the gene *MYO5B*. Then, we performed targeted DNA resequencing of *MYO5B* regions in the large six-generation NSCL/P family to investigate the association of the most important genetic variations and SNP-SNP interactions that may contribute to NSCL/P disease etiology. Further, we performed sequenom iPLEX genotyping on the SNPs that we found significant within the 18q21.1 region by exome sequencing to validate in our larger subset of familial NSCL/P subjects.

Our results identified SNPs within several genes in the 18q21.1 region as potentially pathogenic variants associated with high risk of NSCL/P in this family.

## Materials and methods

### Study subjects

**Figure 1:**
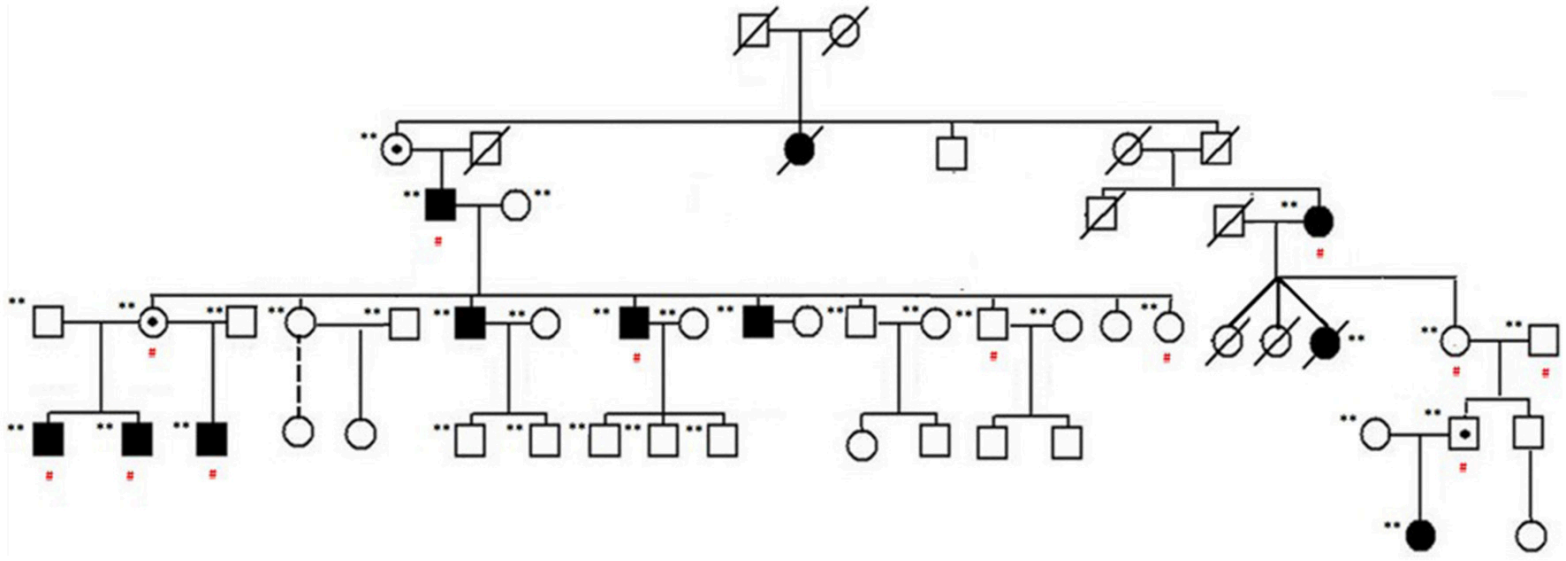
**NSCL/P family pedigree**. Affected individuals are shown with blackened symbols, and unaffected individuals are shown with open symbols. Samples included in the analysis are marked with (^**^) beside their pedigree symbols. A dot in the center of a symbol indicates an individual who is an obligate carrier and produced affected children with NSCL/P. Red (#) indicates those individuals exome sequenced as part of this study.

Familial NSCL/P subjects included in the study are shown in the pedigree provided in Figure 1. Affected individuals are shown with blackened symbols, and unaffected individuals are shown with open symbols. A dot in the center of a symbol denotes an individual who is an obligate carrier and produced affected children with NSCL/P. Samples included in the analysis are marked with (^**^) beside the pedigree symbols. Red (#) indicates individuals who were exome sequenced. The study was approved by the Institutional Review Board at the University of Minnesota.

### DNA isolation and exome sequencing

High-quality DNA was isolated from peripheral blood samples obtained after informed consent from the family members of the six-generation family (*n* = 33: 10 affected + 4 obligate carriers + 19 unaffected relatives) using DNA-extraction kits, described previously (Beiraghi et al., 2007).

Exome sequencing was performed on 12 genomes (six affected individuals, two obligate carriers, and four unaffected individuals) from the NSCL/P family using Illumina HiSeq with TruSeq Exome Enrichment (Illumina, Inc., San Diego, CA, USA).

### Exome analysis pipeline

High-quality, binary alignment mapping (.bam) files were generated by processing raw reads as described in the PALEOMIX mapping pipeline (Supplementary Figure 1) which independently processes and then combines both single and paired end data (Li et al., 2009). Briefly, reads were filtered for poor base call quality and adapter contamination using Adapter Removal (Lindgreen, 2012). Filtered reads were mapped to the HG19 human reference genome using Burrows Wheeler Aligner 0.5.9 (BWA) (Li and Durbin, 2009). PCR duplicates were removed and realignment was performed across detected INDELs resulting in 31–54 million high-quality reads per sample. Variants were called using both SAMtools and Genome Analysis Toolkit (GATK) Unified Genotyper for all sites with >8 reads (Li et al., 2009; McKenna et al., 2010). Depth of coverage was calculated using the coverage command in bedtools version 2.25.0 with the features targeted by the TruSeq Exome Enrichment platform. High quality variants were used as markers in a GWAS study associating SNPs to the NSCL/P trait using the PLINK whole genome association analysis toolset.

### Variant filtering

100 Western European (CEU) genomes from the 1000 Genomes Project were utilized as unaffected controls (1000 Genomes Project Consortium et al., 2012). Low quality variants (QUAL < 50) called by GATK were removed and only the intersecting variants between SAMtools and GATK were retained. Only variants found in ≥6 affected individuals and ≤ 2 unaffected individuals were included. Further, using the 1000 Genomes data, variants with a minor allele frequency (MAF) >0.01 (1%) were also removed. Subsequently, we annotated the variants using the human reference database (GRCh37.75) with CADD, PolyPhen, and snpEFF to identify the most likely destructive variants using the following criteria: top 0.5% by CADD (scaled CADD score > 25) and “HIGH” designation by snpEFF (highly destructive effect predicted) or a high confidence PolyPhen (damaging prediction). In addition to the functional and clinical annotation (ClinVar), for each variant, we also gathered information such as the corresponding MAF in the 1000 genomes panel, the deleterious effect prediction by dbSNP, and its association with phenotype (odds ratio) (1000 Genomes Project Consortium et al., 2012; Cingolani et al., 2012; Kircher et al., 2014; Landrum et al., 2014).

### Primer designing and DNA sequencing

Primers were designed for PCR amplification of two separate regions within the *MYO5B* gene at chr18:47349559–47350124 (566 bp) and chr18:47365313–47365672 (360 bp). Primer designing was done using the PrimerSelect module of DNASTAR Lasergene 11 Core Suite software (DNASTAR Inc., Madison, WI) and oligos were synthesized at the University of Minnesota Genomics Center (UMGC). Prior to oligo synthesis, the primer sequences were verified using DNA BLAT and *In-Silico* PCR tools available at the UCSC Genome Browser website (https://genome.ucsc.edu/index.html) to avoid any nonspecific DNA binding. PCR was performed in a 1X PCR buffer using 100 ng of genomic DNA, 10 pmol each of forward and reverse primers, and GoTaq® Colorless Master Mix (Promega Corporation, Madison, WI, USA). Unincorporated nucleotides and primers were removed prior to sequencing through incubation with shrimp alkaline phosphatase and exonuclease I (Affymetrix, Santa Clara, CA, USA) for 30 min at 37°C and inactivation at 80°C for 15 min. Bi-directional DNA Sequencing was performed with an ABI Prism 3700 automated sequencer (Applied Biosystems, Foster City, CA) at the UMGC using the PCR primers (forward and reverse) or internal primers (sequence available on request). Sequences were assembled using SeqMan, the Multiple Sequence Alignment module of DNASTAR Lasergene 11 Core Suite software (DNASTAR Inc., Madison, WI).

### SNP genotyping

Table 1 provides a detailed list of the SNP panel selected for sequenom genotyping along with SNP inclusion criteria. A total of 29 variants from 15 genes located within the chromosome 18q21.1 locus were genotyped in the NSCL/P DNA samples using Sequenom iPLEX genotyping platform that uses MALDI-TOF (matrix-assisted laser desorption ionization-time-of-flight mass spectrometer)-based chemistry. Criteria for SNP selection included MAF < 0.1 in 1000 Genomes project and Odds Ratio ≤ 0.5 or ≥3 from our exome sequencing data analysis.

**Table 1:** **List of SNPs selected for Sequenom iPLEX genotyping**.

**Sl. No**.	**Variation name**	**Location (bp)**	**Gene**
1	rs959655	chr18:52352494	DCC
2	rs728683	chr18:51582627	
3	rs8097060	chr18:50458806	
4	rs6507992	chr18:50382186	SKA1
5	rs11555886	chr18:50286217	CXXC1
6	rs17715416	chr18:49962255	MYO5B
7	rs17713847	chr18:49849792	SCARNA17
8	rs183559995	chr18:49839074	MYO5B
9	rs78201339	chr18:49823406	MYO5B
10	rs34474737	chr18:49562285	LIPG
11	rs3764482	chr18:48942576	SMAD7
12	rs78950893	chr18:48942348	SMAD7
13	rs8091995	chr18:48862698	CTIF
14	rs6507872	chr18:48862073	CTIF
15	rs11082655	chr18:48149234	ZBTB7C
16	rs1787187	chr18:47841113	SMAD2
17	rs1792666	chr18:47836843	SMAD2
18	rs1981	chr18:47834620	SMAD2
19	rs2510019	chr18:47028941	TCEB3B
20	rs328145	chr18:46593486	LOXHD1
21	rs435770	chr18:46571932	LOXHD1
22	rs17690358	chr18:46559258	LOXHD1
23	rs1450425	chr18:46529070	LOXHD1
24	rs188269968	chr18:46518017	LOXHD1
25	rs8095374	chr18:46213522	C18orf25
26	rs28699609	chr18:46094913	ATP5A1
27	rs2298787	chr18:46090014	ATP5A1
28	rs8092674	chr18:46086016	ATP5A1
29	rs10468858	chr18:45997899	PSTPIP2

Chromosomal locations are based on human genome hg19 (GRCh37) Assembly.

### Genotype-phenotype association analysis

Genotype and allele frequencies were calculated and SNP data was analyzed for association with NSCL/P risk using a combination of the softwares Haploview 4.2 and snpStats using gender as a covariate. SNPStats is a software application that performs genotype-phenotype association analysis based on linear or logistic regression according to the response variable and calculates raw and adjusted odds ratios along with corresponding 95% confidence intervals (Sole et al., 2006). All statistical tests were two-sided; *p* < 0.05 was used as level of significance.

## Results

### Exome sequencing identified high risk variants within genes in 18q21.1 region

Exome sequencing was used to examine expressed portions of 12 familial genomes. Raw exome reads were sequenced and mapped to the hg19 human reference genome using a protocol targeting high-quality mapping confirmation in individuals prior to variant discovery. Regions targeted by exome sequencing averaged 38X coverage indicating sufficient read depth to accurately discover SNPs. Variants were called using both SAMtools (862,091 variants) as well as GATK (2,174,723 variants) in order to assess consensus between the two callers and to account for possible differences due to arbitrary program parameters. A total of 788,916 high-quality variants were in the intersection between GATK and SAMtools which were prioritized and kept for subsequent analysis. Seven hundred and forty seven variants were within chr18q21.1 region, called by both Broad's GATK (864 variants) and SAMtools (1643 variants). Among these variants, 200 SNPs remained after genotype quality control (GATK QUAL > 50 and sample coverage rate >50%; see Materials and Methods Section). This set of variants were computationally annotated by using the human database GRCh37.75 with snpEff/SnpSif (Cingolani et al., 2012) and tested for association with NSCL/P. Supplementary Table 1 lists all variants in the 18q21 region that appear at sufficient frequency in this family (at least six family members have the alternative allele regardless of their CLP status) regardless of their 1000 genomes allele frequency. SNPs were sorted based on their estimated odds ratio given the affected/unaffected distinction, with those conferring the highest risk at the top. Nearly 20% (40 out of 200) of top SNPs associated with NSCL/P risk in this family within the previously described 18q21.1 locus belonged to the gene *MYO5B*. Other major genes at 18q21.1 that contained high-risk variants include *SMAD7, LOXHD1, SKA1*, and *SCARNA17*.

### Targeted resequencing and fine mapping of *MYO5B* regions

To further characterize the *MYO5B* locus, targeted bidirectional Sanger DNA re-sequencing was performed for the *MYO5B* gene regions harboring the four high-risk variants, chr18:47349559+47350124 (contains the SNPs rs75335611, rs117972198, rs372605995) and chr18:47365313+47365672 (contains rs183559995) on the larger subset of all 33 family members from the six-generation family. A total of 71 genetic variants were identified including 9 indels (insertion-deletions) and 15 SNPs already reported in dbSNP database (Table 2). Eighteen SNPs had minor allele frequencies >25%. Among the variants that were found to be significant in exome sequencing, the SNPs rs75335611, rs117972198, rs372605995 did not significantly segregate with either the affected or unaffected state. Whereas, variant 4 (chr18: 47365444, shown below), which has been reported in dbSNP database (rs183559995) at a population frequency of 0.017 was significantly associated with the affected phenotype. Compared to the GG genotype used as reference, the heterozygous rs183559995 GA genotype had an odds ratio (OR_GG vs. GA_) of 18.09 [95% Confidence Interval (CI) 1.86–176.34; gender-adjusted *p* = 0.0019]. Furthermore, analysis of SNP-SNP interactions showed statistically significant (Wilcoxon *p* < 0.05) NSCL/P risk due to the combined effects of the mutant genotype of rs183559995 (GA) and mutant genotype of any of the following *MYO5B* SNPs rs201748833, rs368561623, rs369480218, rs373003146, rs375226833, rs375530149, or rs75335611 (data not shown).

**Table 2:** **List of dbSNPs identified using DNA re-sequencing of *MYO5B* regions**.

**Sl. No**.	**SNP ID**	**chr:position (bp)**	**Alleles**	**Consequence to transcript**
1	rs112057683	chr18:49823471	G/A	intron_variant/ NMD_transcript_variant/ 3_prime_UTR_variant
2	rs113215300	chr18:49823369	G/T	intron_variant/ NMD_transcript_variant/ 3_prime_UTR_variant
3	rs115116077	chr18:49823552	G/A	intron_variant/ NMD_transcript_variant/ 3_prime_UTR_variant
4	rs116888891	chr18:49823561	C/A	intron_variant/ NMD_transcript_variant/ 3_prime_UTR_variant
5	rs144518115	chr18:49823548	T/C	intron_variant/ NMD_transcript_variant/ 3_prime_UTR_variant
6	rs148796775	chr18:49823549	G/A	intron_variant/ NMD_transcript_variant/ 3_prime_UTR_variant
7	rs201748833	chr18:49823258	T/C	intron_variant/ NMD_transcript_variant/ 3_prime_UTR_variant
8	rs368561623	chr18:49823263	A/G	intron_variant/ NMD_transcript_variant/ 3_prime_UTR_variant
9	rs369480218	chr18:49823644	T/C	intron_variant/ NMD_transcript_variant/ 3_prime_UTR_variant
10	rs372278198	chr18:49823313	C/T	intron_variant/ NMD_transcript_variant/ 3_prime_UTR_variant
11	rs373003146	chr18:49823646	A/T	intron_variant/ NMD_transcript_variant/ 3_prime_UTR_variant
12	rs375226833	chr18:49823643	C/A	intron_variant/ NMD_transcript_variant/ 3_prime_UTR_variant
13	rs375530149	chr18:49823314	C/G	intron_variant/ NMD_transcript_variant/ 3_prime_UTR_variant
14	rs75335611	chr18:49823283	C/T	intron_variant/ NMD_transcript_variant/ 3_prime_UTR_variant
15	rs183559995	chr18:49839074	G/A	upstream_gene_variant/ intron_variant

Chromosomal locations are based on human genome hg19 (GRCh37) Assembly.

### Bioinformatics analysis of rs183559995 (*MYO5B*)

Due to proximity of the *MYO5B* SNP rs183559995 to the exon/intron junction, we used the web-based splice site prediction software Exonic splicing enhancer (ESE) finder to predict whether the mutant allele disrupts the binding of splice site proteins. ESE finder screens for the potential splice sites and binding affinities for the four main serine/arginine (SR)-rich splicing factors (SRSFs): SF2/ASF, SC35, SRp40, and SRp55, (Cartegni et al., 2003). Compared to the wild type allele (A), the mutant allele (G) showed loss of binding site for SRSF1 (IgM-BRCA1) and gain in SRSF2 and SRSF6 binding sites. No change was observed for the binding site of splicing factor SRSF5.

### SNP genotyping and genotype-phenotype association analysis

Table 3 provides results from analysis of association between SNPs found present in Sequenom iPLEX genotyping assay with the risk of NSCL/P in the family. Estimation of *q*-values, the false discovery rate (FDR)-based measure of significance for multiple hypothesis tests, was performed using Bioconductor's *q*-value package in R version 3.2.3 (https://cran.r-project.org/) (Storey, 2002). The detailed results for the significantly associated SNPs, including genotype and allele frequencies (Table 4) and results from genotype-phenotype association analysis between SNPs vs. NSCL/P risk, represented in terms of odds ratios of mutant genotypes (Table 5), were obtained using snpStats software. Results from the analysis of association of candidate variants genotyped in this larger set of NSCL/P family samples (*n* = 33) showed significant risks associated with the mutant genotypes of rs1450425 (*LOXHD1*), rs6507992 (*SKA1*), rs78950893 (*SMAD7*), rs8097060, rs17713847 (*SCARNA17*), rs6507872 (*CTIF*), rs8091995 (*CTIF*), rs183559995 (*MYO5B*), rs17715416 (*MYO5B*). The SNP rs78950893 within *SMAD7* gene showed the highest association with NSCL/P phenotype. Compared to the reference genotype rs78950893 CC, the mutant genotypes combined (CT+TT) presented an OR of 22.69 (95% CI = 2.19–234.94; gender-adjusted *p* = 0.001). The *SKA1* rs6507992 GG genotype displayed a very high OR of 15.41 (95% CI = 1.32–179.97; gender-adjusted *p* = 0.013) when compared to the genotypes rs6507992 AA+GA combined. The SNP rs8097060, located within a gene desert in chromosome 18q21.1 and flanked by the genes *SKA1* and *MAPK4*, also showed high risk association. When combined, the genotypes rs8097060 AG and AA had an OR of 11.27 (95% CI = 1.17–108.20; gender-adjusted *p* = 0.0110) compared to the reference genotype (GG). On the other hand, the *LOXHD1* SNP rs1450425 showed an inverse association. Presence of the heterozygous rs1450425 CT genotype had reduced NSCL/P risk compared to the reference rs1450425 CC genotype (OR = 0.09; 95%CI = 0.01–0.95; gender-adjusted *p* = 0.017). Additionally the SNPs rs17713847 (*SCARNA17*), rs17715416 (*MYO5B*) and the *CTIF* SNPs rs6507872 and rs8091995 showed significant association (*p* < 0.05) with NSCL/P when log-additive models were considered.

**Table 3:** **Results from analysis of association between SNPs genotyped vs. NSCL/P risk in the family**.

**SNP ID**	**Gene name**	**Associated allele**	**Chi square**	***q*-value**
rs2298787	ATP5A1	T	1.537	0.173
rs28699609	ATP5A1	A	1.537	0.173
rs8092674	ATP5A1	C	1.133	0.191
rs8095374	C18orf25	T	1.164	0.191
rs6507872	CTIF	T	4.592	0.045
rs8091995	CTIF	T	4.592	0.045
rs11555886	CXXC1	C	0.529	0.293
rs959655	DCC	G	0.166	0.307
rs34474737	LIPG	T	0.283	0.297
rs188269968	LOXHD1	T	12.060	0.006
rs1450425	LOXHD1	C	3.094	0.089
rs435770	LOXHD1	C	1.248	0.191
rs17690358	LOXHD1	C	0.388	0.297
rs328145	LOXHD1	C	0.269	0.297
rs17715416	MYO5B	G	5.770	0.045
rs183559995	MYO5B	A	5.070	0.045
rs78201339	MYO5B	G	0.001	0.391
rs10468858	PSTPIP2	C	0.162	0.307
rs17713847	SCARNA17	A	6.371	0.045
rs6507992	SKA1	G	6.207	0.045
rs1792666	SMAD2	T	0.141	0.307
rs1981	SMAD2	G	0.060	0.337
rs78950893	SMAD7	T	5.070	0.045
rs3764482	SMAD7	A	2.012	0.147
rs2510019	TCEB3B	C	3.148	0.089
rs11082655	ZBTB7C	G	0.320	0.297
rs8097060		A	2.560	0.112
rs728683		A	0.422	0.297

**Table 4:** **Details of allele and genotype frequencies of the significant SNPs genotyped in NSCL/P family members obtained using snpStats**.

**SNP**	**Gene**	**Alleles (*n* = 66)**			**Genotypes (*n* = 33)**		
			**Control**	**NSCL/P**		**Control**	**NSCL/P**
		**Allele**	**Count (Proportion)**	**Count (Proportion)**	**Genotype**	**Count (Proportion)**	**Count (Proportion)**
rs1450425	*LOXHD1*	C	33 (0.75)	17 (0.94)	C/C	11 (0.5)	8 (0.89)
		T	11 (0.25)	1 (0.06)	C/T	11 (0.5)	1 (0.11)
rs6507992	*SKA1*	A	30 (0.68)	7 (0.35)	A/A	9 (0.41)	1 (0.1)
		G	14 (0.32)	13 (0.65)	A/G	12 (0.55)	5 (0.5)
					G/G	1 (0.05)	4 (0.4)
rs78950893	*SMAD7*	C	36 (0.82)	11 (0.55)	C/C	15 (0.68)	1 (0.1)
		T	8 (0.18)	9 (0.45)	C/T	6 (0.27)	9 (0.9)
					T/T	1 (0.05)	0 (0)
rs183559995	*MYO5B*	G	38 (0.83)	11 (0.55)	G/A	8 (0.35)	9 (0.9)
		A	8 (0.17)	9 (0.45)	G/G	15 (0.65)	1 (0.1)
rs8097060		G	33 (0.75)	11 (0.55)	A/A	1 (0.05)	0 (0)
		A	11 (0.25)	9 (0.45)	G/A	9 (0.41)	9 (0.9)
					G/G	12 (0.55)	1 (0.1)
rs17713847	*SCARNA17*	G	30 (0.68)	6 (0.33)	A/A	4 (0.18)	3 (0.33)
		A	14 (0.32)	12 (0.67)	G/A	6 (0.27)	6 (0.67)
					G/G	12 (0.55)	0 (0)
rs17715416	*MYO5B*	A	27 (0.61)	5 (0.28)	A/A	9 (0.41)	0 (0)
		G	17 (0.39)	13 (0.72)	A/G	9 (0.41)	5 (0.56)
					G/G	4 (0.18)	4 (0.44)
rs6507872	*CTIF*	C	32 (0.73)	9 (0.45)	C/C	11 (0.5)	0 (0)
		T	12 (0.27)	11 (0.55)	C/T	10 (0.45)	9 (0.9)
					T/T	1 (0.05)	1 (0.1)
rs8091995	*CTIF*	G	32 (0.73)	9 (0.45)	G/G	11 (0.5)	0 (0)
		T	12 (0.27)	11 (0.55)	G/T	10 (0.45)	9 (0.9)
					T/T	1 (0.05)	1 (0.1)

**Table 5:** **Results for the SNPs found significant using snpStats in the genotype-phenotype association analysis between SNPs vs. NSCL/P risk, represented in terms of odds ratios of mutant genotypes**.

				**Association with NSCL/P**			
				**Crude analysis**		**Adjusted by sex**	
**SNP**	**Gene**	**Model**	**Genotype**	**OR (95% CI)**	***P*-value**	**OR (95% CI)**	***P*-value**
rs1450425	*LOXHD1*		C/C	1	0.0320	1	0.0170
			C/T	0.13 (0.01–1.17)		0.09 (0.01–0.95)	
rs6507992	*SKA1*	Recessive	A/A-G/A	1	0.0130	1	0.0130
			G/G	14.00 (1.31–150.03)		15.41 (1.32–179.97)	
		Log-additive	—	6.10 (1.30–28.58)	0.0065	6.68 (1.35–32.94)	0.0054
rs78950893	*SMAD7*	Dominant	C/C	1	0.0013	1	0.0010
			T/C-T/T	19.29 (2.03–183.42)		22.69 (2.19–234.94)	
		Log-additive	—	7.09 (1.30–38.67)	0.0099	8.48 (1.47–48.84)	0.0062
rs183559995	*MYO5B*		G/G	1	0.0021	1	0.0019
			G/A	16.87 (1.80–158.06)		18.09 (1.86–176.34)	
rs8097060		Dominant	G/G	1	0.0110	1	0.0110
			A/G-A/A	10.80 (1.16–100.43)		11.27 (1.17–108.20)	
		Log-additive	—	4.53 (0.87–23.62)	0.0480	4.41 (0.82–23.62)	0.0560
rs17713847	*SCARNA17*	Log-additive	—	3.66 (1.11–12.01)	0.0190	4.77 (1.26–18.09)	0.0089
rs17715416	*MYO5B*	Log-additive	—	4.11 (1.12–15.12)	0.0180	6.14 (1.29–29.30)	0.0065
rs6507872	*CTIF*	Log-additive	—	9.39 (1.16–75.97)	0.0070	17.62 (1.80–172.46)	0.0015
rs8091995	*CTIF*	Log-additive	—	9.39 (1.16–75.97)	0.0070	17.62 (1.80–172.46)	0.0015

## Discussion

Genetic variations have long been considered involved in the risk of syndromic and nonsyndromic CL/P. Mutations in a number of genes have shown promising associations including transcription factors (*IRF6, MSX1, TBX22*), growth factors (*TGFA, TGFb3*), xenobiotic metabolism genes (*CYP1A1, GSTM1, NAT2*), and genes involved in immune response (*PVRL1*), although the results have been conflicting (Ardinger et al., 1989; Hecht et al., 1991; Chenevix-Trench et al., 1992; Vintiner et al., 1992, 1993; Stein et al., 1995; Wyszynski et al., 1997; Lidral et al., 1998; Martinelli et al., 1998; Vieira et al., 2005; Alkuraya et al., 2006; Kerameddin et al., 2015). In addition, recent genomewide association studies have identified 13 different chromosomal loci that may harbor common variants associated with increased risk of NSCL/P including 1p22, 1p36, 2p21, 3p11.1, 8q21.3, 8q24, 9q22, 10q25, 15q22, 17p13, 17q22, and 20q12 (Birnbaum et al., 2009; Grant et al., 2009; Marazita et al., 2009; Beaty et al., 2010; Mangold et al., 2010; Ludwig et al., 2012; Leslie et al., 2015).

However, despite the progress in gene identification for NSCL/P, a reliable high risk mutation signature that underline the mechanisms behind the development of NSCL/P have yet to be identified.

In a previous study, we used SNP array to perform genome-wide linkage analysis on DNA isolated from peripheral blood samples from a large multigenerational family of self-reported European origin to investigate the role of genetic variants toward NSCL/P risk (Beiraghi et al., 2007). The SNP array (GeneChip Mapping 10K *Xba*I Array) consisted of 10,555 SNPs equally distributed in the genome, with mean intermarker distances of 250 kb and an average heterozygosity of 0.38. Our genome wide genotyping study identified a 5.7-Mb genomic region on chromosome 18q21.1 spanned by proximal marker rs1824683 (42,403,918 bp) and distal marker rs768206 (48,132,862 bp) that potentially contains a pathogenic, high-risk variant signature associated with NSCL/P in this family (Beiraghi et al., 2007).

In the current study, we performed fine-mapping of the 18q21.1 region using exome sequencing to identify novel rare pathogenic variants significantly associated with NSCL/P risk. Among the SNPs that conferred the highest risk in exome sequencing, a large number of top candidate variants belonged to the gene *MYO5B* (Chr18: 47349155–47721451), a myosin family member which is involved in protein trafficking, neuronal morphogenesis, cell signaling, vesicular trafficking, plasma membrane recycling and epithelial polarization. Mutations in the *MYO5B* gene have been previously implicated in human diseases including microvillus inclusion disease (MVID) in newborns (Knowles et al., 2014).

Subsequently, targeted re-sequencing of these high-risk *MYO5B* gene regions provided strong evidence that the SNP rs183559995 (G/A) in *MYO5B* is a strong candidate genetic risk variant for NSCL/P in this family. Although, rs183559995 is an intronic variant whose function has not been described, predictions using ESEFinder indicate that presence of the mutant allele (A) has the potential to disrupt binding of splicing factors. Further, SNP-SNP interaction analysis showed statistically significant increase in NSCL/P risk due to the combined effects of the presence of rs183559995 (A) along with mutant alleles of these *MYO5B* SNPs rs201748833, rs368561623, rs369480218, rs373003146, rs375226833, rs375530149, or rs75335611. In addition, we also found another *MYO5B* SNP, rs17715416 that showed significant association with NSCL/P (gender-adjusted P_log−additive model_ = 0.0065).

**Figure 2:**
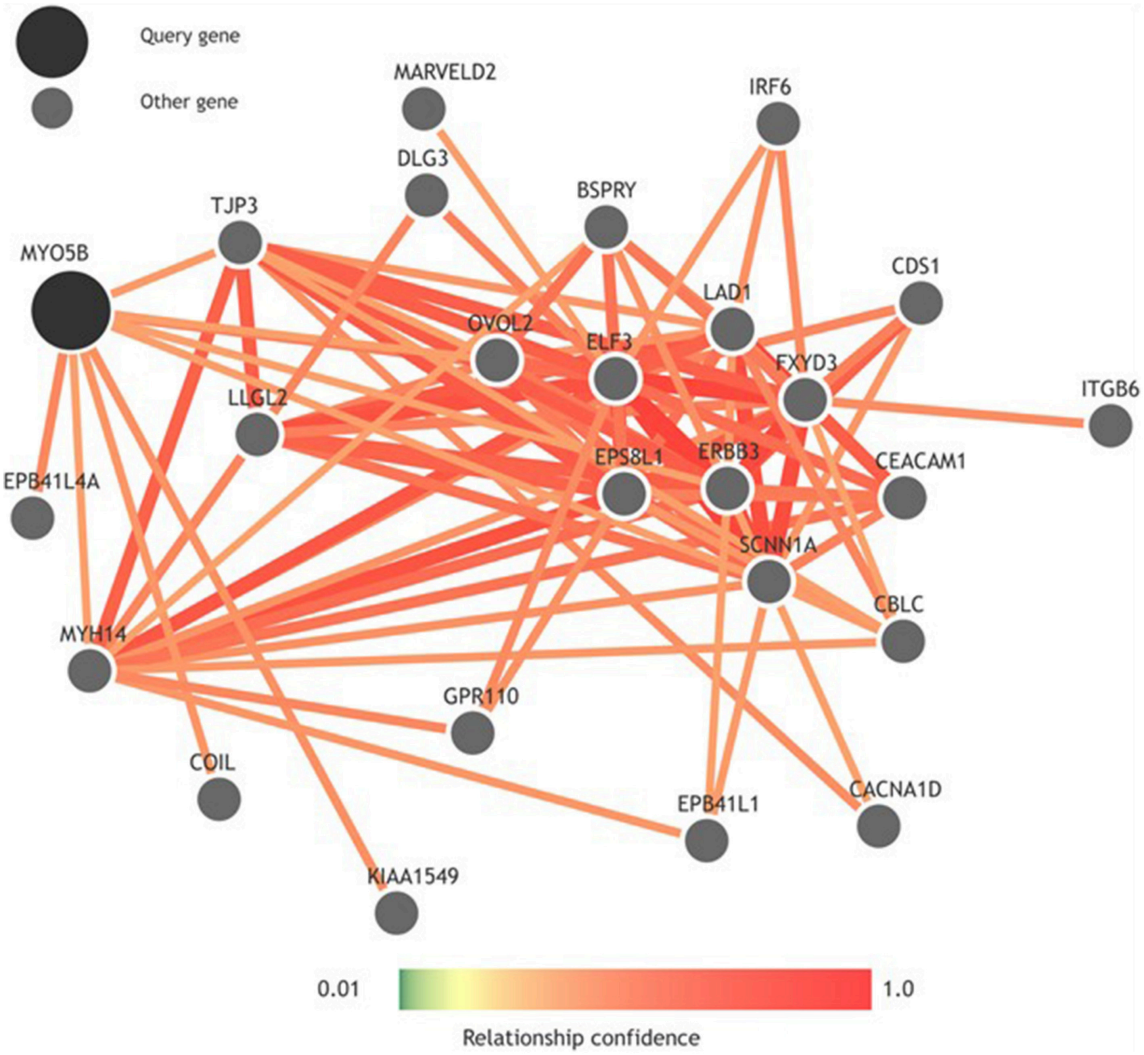
**Functional network built using GIANT (Genome-scale Integrated Analysis of gene Networks in Tissues) showing neighbors of MYO5B**.

To look more into the interactions of the *MYO5B* gene, we used GIANT (Genome-scale Integrated Analysis of gene Networks in Tissues), a webserver-based system that integrates human genomic data to build functional networks with edges supported by various types of interaction or co-expression evidence (Greene et al., 2015). Interestingly, while looking into the neighbors of *MYO5B* using GIANT (Figure 2), we observed a network containing a set of functional neighbors for *MYO5B* is enriched for the KEGG “Epithelial tight junction” pathway. This was mainly driven by its similarity to members of this pathway including *TJP3, MYH14, EPB41L1, LLGL2* suggesting that *MYO5B* may play a role here. This seems to have some support from more focused studies as well. For instance, an earlier study on MVID—a form of congenital enteropathy, indicates that the expression of MYO5B-P660L (an MVID-associated mutation found within Navajo populations) in patients with MVID resulted in global changes in polarity at the villus tips that could lead to a number of complications including aberrant junctions, and losses in transcellular ion transport pathways (Knowles et al., 2014). It is of considerable interest to note that epithelial tight junctions are of relevance to cleft lip/palate and that aberrant junctions have been previously implicated in NSCL/P. For example, mutations in *PVRL1* (nectin-1), which plays a key role in adherens junctions, have previously been associated with cleft lip/palate (Sozen et al., 2001). This combination of evidence strengthens the potential connection between the *MYO5B* mutations and tight junctions, which might eventually influence NSCL/P risk. Additional studies will be required to determine significance with regard to *MYO5B* structure and function in NSCL/P pathology.

Furthermore, we performed genotyping of 29 SNPs included from the 18q21.1 region to identify additional genetic variants associated with NSCL/P risk. The *p*-values from logistic regression analysis were gender-adjusted to account for the gender-based differences in prevalence. SNP genotyping studies found the mutant genotypes of the following SNPs were associated with NSCL/P risk: rs78950893 (*SMAD7*), rs1450425 (*LOXHD1*), rs6507992 (*SKA1*), rs8097060, rs17713847 (*SCARNA17*), rs6507872 (*CTIF*), rs8091995 (*CTIF*), and rs17715416 (*MYO5B*). Inhibition of SMAD pathway by all-trans retinoic acid (atRA) have previously been implicated in cleft palate. It was shown that atRA-induced inhibition of SMAD pathway played important role in the degradation of the basal laminin within the midline epithelial seam (MES) which might contribute to failure of palatal fusion (Wang et al., 2011). In our study, the combined mutant genotype (CT+TT) of the *SMAD7* SNP rs78950893 showed the highest association with NSCL/P phenotype (OR_CT+TT vs. CC_ = 22.69; 95% CI = 2.19–234.94; gender-adjusted *p* = 0.001). In contrast, the heterozygous *LOXHD1* rs1450425 CT genotype was found positively associated with NSCL/P risk. *LOXHD1* codes for a highly conserved conserved stereociliary protein involved in targeting proteins to the plasma membrane. LOXHD1 mutations have been previously implicated in the genetic etiology of autosomal recessive nonsyndromic hearing loss (ARNSHL) (Atik et al., 2015). Among the other genes found significant, spindle- and kinetochore-associated protein 1 (*SKA1*) is a microtubule-binding protein that localizes to spindle microtubule and the outer kinetochore interface during mitosis and is therefore essential for proper chromosome segregation (Li et al., 2014). In our study, *SKA1* SNP rs6507992 showed highly significant association with the NSCL/P phenotype, rs6507992 GG had an odds ratio of 15.41 compared to the rs6507992 AA and GA genotypes combined (OR_AA+GA vs. GG_ = 15.41; 95% CI = 1.32–179.97; gender-adjusted *p* = 0.0130). CBP80/20-dependent translation initiation factor (*CTIF*) is a component of the CBP80 translation initiation complex that binds cotranscriptionally to the cap end of nascent mRNA, recognizes premature termination codons (PTCs) in mRNAs and directs nonsense-mediated decay (NMD) in PTC-containing mRNAs. SNPs in *CTIF* have been shown to be associated with hearing function in children (Harrison et al., 2015). We found two SNPs in *CTIF* gene (rs6507872 and rs8091995) with statistically significant association with NSCL/P risk (gender-adjusted P_log−additive model_ = 0.0015, for both).

Exome sequencing of this family showed several loci with co-segregating variants associated with NSCP/P. This relatively small cohort of 12 individuals, even when cross referencing variants observed within the 1000 genomes, poses several difficulties. First, the low penetrance of the disease confounds case/control status for several of the individuals within the family (Figure 1). Second, targeted exome sequencing assumes adequate depth of coverage to detect causal mutations and, additionally, does not account for larger structural variation such as insertions or deletions that could potentially be causing the phenotype. However, given the number of SNPs which had high odds ratios in the gene MYO5B, fine mapping through targeted resequencing was performed of this region in a larger familial cohort to supplement variants discovered by whole exome sequencing. Using fine-mapping of the chromosome 18q21.1 region, we could identify SNPs that are strong candidates for association with familial NSCL/P risk. Further studies are required in terms of linkage disequilibrium analysis and functional genomics to determine the extent of significance of these high risk variants vis-à-vis gene function and role in the complex genetic etiology of NSCL/P. Moreover, low penetrance of NSCL/P in this family suggests a multigenic model which will require identification of additional variants, analysis of potential copy number variation (CNV) burden from the exome data and further studies in larger sets of families/pedigrees to derive a robust high-risk SNP signature profile associated with NSCL/P.

## Author contributions

AM designed experimental procedures and conducted experiments, performed data analysis and wrote most of the manuscript. HS contributed to experiment design, sample processing, and manuscript writing. RS, WW, and CM provided technical computation support in Exome sequencing data analysis and manuscript writing. BV and SB supervised all project design.

### Conflict of interest statement

The authors declare that the research was conducted in the absence of any commercial or financial relationships that could be construed as a potential conflict of interest.
