# Supplementary material for: Fine-Mapping of 18q21.1 Locus Identifies Single Nucleotide Polymorphisms Associated with Nonsyndromic Cleft Lip with or without Cleft Palate
Source: Front Genet. 2016 May 23;7:88. doi: 10.3389/fgene.2016.00088 (PMC4876112; doi:10.3389/fgene.2016.00088)
Supplement: Supplementary file 1 [file Table1.DOCX]

**Supplementary Table 1:** List of SNPs within 18q21.1 region found in exome sequencing following SNP filtering

All variants were annotated using human reference database (GRCh37.75) to identify the most likely destructive variants using the following criteria: top 0.5% by CADD (scaled CADD score > 25) and “HIGH” designation by snpEFF (highly destructive effect predicted) or a high confidence PolyPhen damaging prediction, and functional and clinical annotation (ClinVar). Minor allele frequency (MAF) in the 1000 Genomes panel and association with phenotype (odds ratio) are also provided.

| **Chromosome** | **Position** | **SNP ID** | **Reference allele** | **Alternate allele** | **Gene** | **Minor allele Frequency (1000Genome project)** | **Odds Ratio** | **SNP EFFECT** |
| --- | --- | --- | --- | --- | --- | --- | --- | --- |
| **chr18** | 46247836 | rs6507863 | C | T | *CTIF* | 0 | 1.000 | DOWNSTREAM |
| **chr18** | 43610235 | rs473371 | T | C | *RP11-8H2.1* | 0 | 0.000 | UTR_3_PRIME |
| **chr18** | 43843736 | rs9956233 | T | G | *C18orf25* | 0 | 0.000 | DOWNSTREAM |
| **chr18** | 45367484 | rs1787187 | A | G | *SMAD2* | 0 | 0.000 | DOWNSTREAM |
| **chr18** | 47365444 | rs183559995 | G | A | *MYO5B* | 0.001377 | 4.000 | INTRON |
| **chr18** | 44635048 | rs2635058 | C | T | *RP11-49K24.8* | 0.0037 | 0.000 | INTRON |
| **chr18** | 44097980 | rs188269968 | G | T | *LOXHD1* | 0.004132 | 3.000 | INTERGENIC |
| **chr18** | 43610037 | rs677892 | A | G | *RP11-8H2.1* | 0.0051 | 0.000 | DOWNSTREAM |
| **chr18** | 45070973 | rs6507767 | C | T |  | 0.0234 | 0.800 | DOWNSTREAM |
| **chr18** | 44114473 | rs436433 | T | C | *LOXHD1* | 0.0275 | 0.000 | INTRON |
| **chr18** | 47563299 | rs1815930 | T | C | *MYO5B* | 0.028 | 0.000 | INTRON |
| **chr18** | 47911837 | rs7234507 | A | T | *SKA1* | 0.0308 | 2.333 | INTERGENIC |
| **chr18** | 45228393 | rs7234140 | T | C |  | 0.0308 | 1.000 | DOWNSTREAM |
| **chr18** | 47901516 | rs6507990 | A | G | *SKA1* | 0.0381 | 0.000 | INTRON |
| **chr18** | 47175990 | rs35864578 | G | A |  | 0.05969 | 0.333 | INTRON |
| **chr18** | 46004809 | rs7231325 | T | C |  | 0.0615 | 0.800 | UTR_5_PRIME |
| **chr18** | 46005756 | rs7235465 | C | T |  | 0.0624 | 0.800 | INTERGENIC |
| **chr18** | 44139221 | rs17690358 | C | T | *LOXHD1* | 0.06474 | 0.333 | EXON |
| **chr18** | 47376162 | rs17713847 | G | A | *SCARNA17* | 0.06703 | 0.800 | EXON |
| **chr18** | 47812587 | rs11555886 | C | T | *CXXC1* | 0.0753 | 0.500 | INTRON |
| **chr18** | 46468718 | rs78950893 | C | T | *SMAD7* | 0.08035 | 4.000 | EXON |
| **chr18** | 44286439 | rs2155987 | A | G | *ST8SIA5* | 0.09 | 4.500 | DOWNSTREAM |
| **chr18** | 44272034 | rs533027 | A | C | *ST8SIA5* | 0.0932 | 0.000 | INTRON |
| **chr18** | 47811875 | rs2276364 | A | G | *CXXC1* | 0.095 | 0.000 | EXON |
| **chr18** | 47810731 | rs2276367 | T | C | *CXXC1* | 0.0973 | 0.000 | DOWNSTREAM |
| **chr18** | 47788256 | rs698617 | C | T | *MBD1* | 0.1042 | 1.500 | DOWNSTREAM |
| **chr18** | 47376128 | rs490697 | A | G | *SCARNA17* | 0.1056 | 0.000 | DOWNSTREAM |
| **chr18** | 47795629 | rs11663629 | C | A | *MBD1* | 0.1061 | 0.000 | EXON |
| **chr18** | 48015300 | rs2958018 | C | G |  | 0.1097 | 0.467 | DOWNSTREAM |
| **chr18** | 46201052 | rs299738 | A | G | *MIR4743* | 0.1102 | 1.000 | INTERGENIC |
| **chr18** | 46468946 | rs3764482 | G | A | *SMAD7* | 0.1129 | 4.000 | DOWNSTREAM |
| **chr18** | 47402280 | rs657424 | A | C | *MYO5B* | 0.1212 | 0.000 | DOWNSTREAM |
| **chr18** | 47814249 | rs8089314 | T | C | *CXXC1* | 0.135 | 0.000 | DOWNSTREAM |
| **chr18** | 43505558 | rs4476262 | C | G | *EPG5* | 0.141 | 0.500 | START_GAINED |
| **chr18** | 46388444 | rs6507872 | C | T | *CTIF* | 0.1451 | 9.000 | DOWNSTREAM |
| **chr18** | 47753469 | rs894770 | C | T | *CCDC11* | 0.1478 | 0.800 | INTRON |
| **chr18** | 46284587 | rs1038308 | T | C | *CTIF* | 0.1497 | 4.500 | DOWNSTREAM |
| **chr18** | 45675605 | rs11082655 | T | G | *ZBTB7C* | 0.1552 | 20.000 | DOWNSTREAM |
| **chr18** | 46389069 | rs8091995 | G | T | *CTIF* | 0.1579 | 9.000 | INTRON |
| **chr18** | 43531365 | rs58312765 | A | C | *EPG5* | 0.1584 | 0.750 | DOWNSTREAM |
| **chr18** | 47985176 | rs8097060 | G | A |  | 0.1754 | 4.000 | DOWNSTREAM |
| **chr18** | 46006243 | rs8094888 | A | G |  | 0.1758 | 0.800 | INTRON |
| **chr18** | 45230173 | rs8098085 | C | T |  | 0.1837 | 1.500 | DOWNSTREAM |
| **chr18** | 44268611 | rs328119 | G | T | *ST8SIA5* | 0.191 | 4.000 | NON_SYNONYMOUS_CODING |
| **chr18** | 46196880 | rs2279304 | T | G | *RP11-426J5.2* | 0.1919 | 4.000 | DOWNSTREAM |
| **chr18** | 47777244 | rs35193847 | C | T | *CCDC11* | 0.2025 | 2.000 | INTRON |
| **chr18** | 47765121 | rs35839946 | G | C | *CCDC11* | 0.2107 | 2.000 | INTRON |
| **chr18** | 43509045 | rs73953914 | A | G | *EPG5* | 0.2117 | 2.000 | DOWNSTREAM |
| **chr18** | 43652203 | rs8085444 | G | A | *Y_RNA* | 0.2117 | 0.500 | INTRON |
| **chr18** | 47778172 | rs35295154 | A | G | *CCDC11* | 0.2126 | 2.000 | DOWNSTREAM |
| **chr18** | 47801800 | rs140690 | G | A | *MBD1* | 0.2176 | 1.167 | INTRON |
| **chr18** | 47906719 | rs1441295 | T | G | *SKA1* | 0.2365 | 2.000 | INTRON |
| **chr18** | 47906690 | rs1441294 | T | C | *SKA1* | 0.2365 | 0.800 | INTRON |
| **chr18** | 47906336 | rs6507991 | T | C | *SKA1* | 0.2365 | 0.750 | INTRON |
| **chr18** | 47906416 | rs1441293 | A | G | *SKA1* | 0.2365 | 0.000 | DOWNSTREAM |
| **chr18** | 47908556 | rs6507992 | G | A | *SKA1* | 0.2383 | 0.200 | INTRON |
| **chr18** | 47902337 | rs1970679 | A | G | *SKA1* | 0.2401 | 0.000 | NON_SYNONYMOUS_CODING |
| **chr18** | 43508800 | rs9965433 | G | A | *EPG5* | 0.2406 | 2.000 | DOWNSTREAM |
| **chr18** | 44173449 | rs328145 | A | C | *LOXHD1* | 0.2452 | 4.500 | INTRON |
| **chr18** | 43698447 | rs1866322 | A | G | *RNU6-1278P* | 0.2557 | 1.000 | UTR_3_PRIME |
| **chr18** | 43844734 | rs9959526 | T | G | *C18orf25* | 0.2562 | 4.000 | DOWNSTREAM |
| **chr18** | 47093790 | rs2000812 | C | T | *LIPG* | 0.2562 | 0.000 | INTRON |
| **chr18** | 43666952 | rs8097521 | C | A | *ATP5A1* | 0.2576 | 0.200 | DOWNSTREAM |
| **chr18** | 47809871 | rs12458654 | G | T | *CXXC1* | 0.2612 | 2.000 | INTRON |
| **chr18** | 47176793 | rs4939886 | A | G |  | 0.27 | 0.000 | INTRON |
| **chr18** | 47088655 | rs34474737 | T | G | *LIPG* | 0.2723 | 0.333 | DOWNSTREAM |
| **chr18** | 43577865 | rs10468858 | G | C | *PSTPIP2* | 0.2764 | 0.333 | DOWNSTREAM |
| **chr18** | 43577908 | rs10468980 | T | C | *PSTPIP2* | 0.2769 | 0.333 | DOWNSTREAM |
| **chr18** | 43669980 | rs2298787 | T | C | *ATP5A1* | 0.2782 | 0.200 | EXON |
| **chr18** | 43674879 | rs28699609 | A | T | *ATP5A1* | 0.2782 | 0.200 | DOWNSTREAM |
| **chr18** | 43667528 | rs7244921 | T | C | *ATP5A1* | 0.2787 | 0.500 | DOWNSTREAM |
| **chr18** | 43664115 | rs12954944 | T | C | *ATP5A1* | 0.2787 | 0.200 | DOWNSTREAM |
| **chr18** | 43665982 | rs8092674 | C | T | *ATP5A1* | 0.2787 | 0.200 | DOWNSTREAM |
| **chr18** | 43668321 | rs11659795 | G | T | *ATP5A1* | 0.2787 | 0.200 | INTRON |
| **chr18** | 47810351 | rs7228084 | A | G | *CXXC1* | 0.2874 | 0.000 | EXON |
| **chr18** | 47488625 | rs17715416 | A | G | *MYO5B* | 0.2925 | 4.500 | INTRON |
| **chr18** | 43793488 | rs8095374 | T | C | *C18orf25* | 0.2966 | 3.000 | DOWNSTREAM |
| **chr18** | 43579395 | rs55966192 | T | C | *PSTPIP2* | 0.3049 | 0.333 | DOWNSTREAM |
| **chr18** | 44126909 | rs1893566 | T | C | *LOXHD1* | 0.3053 | 0.000 | DOWNSTREAM |
| **chr18** | 43568593 | rs3844540 | T | G | *RN7SKP26* | 0.309 | 0.333 | DOWNSTREAM |
| **chr18** | 43570777 | rs7242970 | C | T | *PSTPIP2* | 0.3095 | 0.333 | INTRON |
| **chr18** | 43585536 | rs9675392 | A | C | *PSTPIP2* | 0.3113 | 0.333 | DOWNSTREAM |
| **chr18** | 47802971 | rs10775495 | A | G | *MBD1* | 0.3113 | 0.000 | DOWNSTREAM |
| **chr18** | 44601611 | rs3816125 | A | G | *RP11-49K24.4* | 0.3118 | 4.500 | DOWNSTREAM |
| **chr18** | 43565195 | rs1048827 | G | T | *RN7SKP26* | 0.3118 | 0.333 | EXON |
| **chr18** | 47984886 | rs980421 | T | C |  | 0.3159 | 4.000 | DOWNSTREAM |
| **chr18** | 43565399 | rs58786055 | C | A | *RN7SKP26* | 0.32 | 0.333 | DOWNSTREAM |
| **chr18** | 43565514 | rs60116181 | G | A | *RN7SKP26* | 0.32 | 0.333 | DOWNSTREAM |
| **chr18** | 43565380 | rs57589400 | A | G | *RN7SKP26* | 0.3205 | 0.333 | DOWNSTREAM |
| **chr18** | 43563472 | rs9630785 | G | A | *PSTPIP2* | 0.3214 | 0.333 | EXON |
| **chr18** | 43564296 | rs7229227 | G | A | *PSTPIP2* | 0.3219 | 0.333 | DOWNSTREAM |
| **chr18** | 43563677 | rs67746112 | T | C | *PSTPIP2* | 0.3223 | 0.333 | EXON |
| **chr18** | 43564506 | rs7229264 | A | G | *PSTPIP2* | 0.3223 | 0.333 | DOWNSTREAM |
| **chr18** | 43568717 | rs2276199 | T | C | *PSTPIP2* | 0.3223 | 0.333 | INTRON |
| **chr18** | 47361598 | rs535045 | C | T | *MYO5B* | 0.3223 | 1.167 | DOWNSTREAM |
| **chr18** | 43523312 | rs3745000 | G | A | *EPG5* | 0.3232 | 2.000 | EXON |
| **chr18** | 47787402 | rs1899671 | G | A | *CCDC11* | 0.3251 | 0.000 | INTRON |
| **chr18** | 47911438 | rs7234010 | C | A | *SKA1* | 0.3255 | 0.800 | INTRON |
| **chr18** | 47802458 | rs140687 | C | A | *MBD1* | 0.331 | 0.000 | INTRON |
| **chr18** | 43608511 | rs16978516 | T | C | *RP11-8H2.1* | 0.3329 | 0.333 | START_GAINED |
| **chr18** | 48190271 | rs3752085 | T | G | *MAPK4* | 0.3329 | 4.500 | DOWNSTREAM |
| **chr18** | 47463872 | rs1787519 | A | G | *MYO5B* | 0.343 | 2.000 | DOWNSTREAM |
| **chr18** | 44702718 | rs2277717 | C | A | *IER3IP1* | 0.3489 | 1.167 | INTRON |
| **chr18** | 47480898 | rs1557359 | T | C | *MYO5B* | 0.3499 | 4.500 | DOWNSTREAM |
| **chr18** | 47350601 | rs607552 | T | G | *MYO5B* | 0.3499 | 0.000 | DOWNSTREAM |
| **chr18** | 44681356 | rs2251948 | A | T | *IER3IP1* | 0.3508 | 2.333 | DOWNSTREAM |
| **chr18** | 44122010 | rs10775482 | T | C | *LOXHD1* | 0.3522 | 0.000 | INTRON |
| **chr18** | 44664304 | rs2684820 | G | A | *RP11-49K24.5* | 0.3522 | 3.000 | DOWNSTREAM |
| **chr18** | 44664131 | rs2635050 | G | A | *RP11-49K24.5* | 0.3526 | 4.500 | INTRON |
| **chr18** | 47363009 | rs621101 | C | T | *MYO5B* | 0.3531 | 0.800 | INTRON |
| **chr18** | 44122540 | rs2365336 | C | G | *LOXHD1* | 0.3535 | 0.300 | DOWNSTREAM |
| **chr18** | 47463587 | rs1790797 | A | G | *MYO5B* | 0.3558 | 4.500 | DOWNSTREAM |
| **chr18** | 47375932 | rs488890 | C | G | *MYO5B* | 0.36 | 0.000 | EXON |
| **chr18** | 47376103 | rs490648 | C | G | *SCARNA17* | 0.36 | 0.000 | INTRON |
| **chr18** | 44174553 | rs328144 | C | T | *LOXHD1* | 0.3691 | 2.000 | DOWNSTREAM |
| **chr18** | 44109115 | rs59252058 | G | A | *LOXHD1* | 0.3701 | 4.500 | INTERGENIC |
| **chr18** | 44109033 | rs1450425 | T | C | *LOXHD1* | 0.3765 | 0.000 | INTRON |
| **chr18** | 44098736 | rs426303 | A | G | *LOXHD1* | 0.3783 | 0.800 | DOWNSTREAM |
| **chr18** | 46689877 | rs7231453 | G | A | *DYM* | 0.3834 | 1.167 | INTRON |
| **chr18** | 45362194 | rs1792671 | T | C | *SMAD2* | 0.3843 | 0.000 | DOWNSTREAM |
| **chr18** | 45360991 | rs1981 | A | G | *SMAD2* | 0.3848 | 0.000 | UTR_3_PRIME |
| **chr18** | 46343818 | rs9951895 | T | C | *CTIF* | 0.3921 | 0.750 | UTR_3_PRIME |
| **chr18** | 46146221 | rs2306514 | G | A | *CTIF* | 0.3949 | 0.300 | INTRON |
| **chr18** | 47166098 | rs4939882 | G | A |  | 0.3972 | 4.500 | DOWNSTREAM |
| **chr18** | 47177814 | rs12456395 | T | C |  | 0.3976 | 3.000 | DOWNSTREAM |
| **chr18** | 47177833 | rs12458748 | C | T |  | 0.3976 | 1.500 | DOWNSTREAM |
| **chr18** | 47810251 | rs3819217 | C | G | *CXXC1* | 0.3976 | 1.167 | DOWNSTREAM |
| **chr18** | 47177724 | rs948938 | G | C |  | 0.3985 | 1.167 | EXON |
| **chr18** | 47177461 | rs948941 | A | C |  | 0.3985 | 0.800 | DOWNSTREAM |
| **chr18** | 47177541 | rs948940 | C | G |  | 0.3985 | 0.800 | EXON |
| **chr18** | 47177311 | rs4939593 | C | T |  | 0.3985 | 0.467 | INTERGENIC |
| **chr18** | 47406913 | rs1217632 | A | G | *MYO5B* | 0.4027 | 0.000 | INTRON |
| **chr18** | 47406938 | rs1217633 | T | C | *MYO5B* | 0.4027 | 0.000 | INTRON |
| **chr18** | 48008659 | rs8089403 | G | A |  | 0.4169 | 4.000 | INTRON |
| **chr18** | 44560429 | rs892586 | C | A | *KATNAL2* | 0.4183 | 0.333 | INTERGENIC |
| **chr18** | 47978199 | rs2919771 | A | G |  | 0.4206 | 9.000 | DOWNSTREAM |
| **chr18** | 47113008 | rs6507931 | C | T | *LIPG* | 0.4224 | 0.800 | DOWNSTREAM |
| **chr18** | 47918639 | rs10460085 | A | C | *SKA1* | 0.4224 | 0.500 | UTR_3_PRIME |
| **chr18** | 45359664 | rs8671 | T | A | *SMAD2* | 0.4229 | 0.000 | DOWNSTREAM |
| **chr18** | 45363214 | rs1792666 | A | T | *SMAD2* | 0.4229 | 0.000 | SYNONYMOUS_CODING |
| **chr18** | 47455923 | rs2298628 | C | T | *MYO5B* | 0.4233 | 0.000 | INTRON |
| **chr18** | 46384073 | rs937021 | A | G | *CTIF* | 0.4247 | 4.500 | INTRON |
| **chr18** | 44426877 | rs644731 | C | T | *PIAS2* | 0.4265 | 4.500 | INTRON |
| **chr18** | 44151895 | rs435770 | T | C | *LOXHD1* | 0.4265 | 2.000 | INTERGENIC |
| **chr18** | 47943851 | rs4939981 | T | C |  | 0.4284 | 0.500 | INTERGENIC |
| **chr18** | 43844658 | rs8086610 | A | C | *C18orf25* | 0.4302 | 0.000 | INTRON |
| **chr18** | 47943773 | rs4939980 | G | A |  | 0.4343 | 0.500 | INTRON |
| **chr18** | 44558912 | rs2576050 | T | C | *TCEB3B* | 0.4343 | 0.333 | UTR_3_PRIME |
| **chr18** | 43845269 | rs3744858 | C | T | *C18orf25* | 0.4353 | 0.000 | DOWNSTREAM |
| **chr18** | 43698309 | rs1866321 | C | T | *RNU6-1278P* | 0.4362 | 0.000 | UTR_3_PRIME |
| **chr18** | 43652345 | rs3745002 | A | G | *Y_RNA* | 0.4417 | 1.500 | DOWNSTREAM |
| **chr18** | 44561100 | rs2571028 | C | G | *KATNAL2* | 0.4458 | 0.333 | DOWNSTREAM |
| **chr18** | 47102002 | rs2276269 | T | C | *LIPG* | 0.4472 | 0.000 | INTRON |
| **chr18** | 45156441 | rs3829617 | A | G |  | 0.4481 | 0.000 | INTRON |
| **chr18** | 46543380 | rs9948469 | T | C |  | 0.4486 | 8.000 | INTERGENIC |
| **chr18** | 47943863 | rs2919788 | T | C |  | 0.4522 | 0.500 | INTERGENIC |
| **chr18** | 47339798 | rs2945506 | G | A | *ACAA2* | 0.4527 | 0.300 | DOWNSTREAM |
| **chr18** | 46385948 | rs3752082 | A | G | *CTIF* | 0.4573 | 4.500 | INTERGENIC |
| **chr18** | 47317767 | rs529556 | C | T | *ACAA2* | 0.4614 | 0.000 | INTRON |
| **chr18** | 47917735 | rs2044012 | A | C | *SKA1* | 0.4633 | 0.500 | INTERGENIC |
| **chr18** | 44595809 | rs2289036 | A | T | *KATNAL2* | 0.4665 | 0.000 | EXON |
| **chr18** | 46387889 | rs3809921 | A | G | *CTIF* | 0.4674 | 4.000 | INTRON |
| **chr18** | 45227494 | rs12606393 | A | G |  | 0.4683 | 4.667 | DOWNSTREAM |
| **chr18** | 47352533 | rs555879 | G | A | *MYO5B* | 0.4715 | 0.000 | DOWNSTREAM |
| **chr18** | 43703223 | rs12954742 | C | G | *HAUS1* | 0.4747 | 0.800 | INTRON |
| **chr18** | 45887180 | rs4335832 | A | C | *ZBTB7C* | 0.4752 | 0.333 | DOWNSTREAM |
| **chr18** | 47176911 | rs4939887 | A | G |  | 0.4766 | 0.000 | EXON |
| **chr18** | 47176917 | rs4939888 | T | C |  | 0.4766 | 0.000 | INTRON |
| **chr18** | 47480660 | rs1787299 | G | A | *MYO5B* | 0.4853 | 4.500 | EXON |
| **chr18** | 44641397 | rs2576058 | C | T | *HDHD2* | 0.4871 | 2.333 | INTRON |
| **chr18** | 44526798 | rs2187092 | A | C | *KATNAL2* | 0.4968 | 0.000 | INTRON |
| **chr18** | 47373466 | rs3017176 | G | A | *MYO5B* | 0.4972 | 0.000 | DOWNSTREAM |
| **chr18** | 47405198 | rs596778 | C | T | *MYO5B* | 0.4977 | 0.800 | INTRON |
| **chr18** | 44702502 | rs12968552 | A | C | *IER3IP1* | 0.4995 | 0.800 | DOWNSTREAM |
| **chr18** | 44555312 | rs76539063 | G | C | *TCEB3B* | NA | 4.500 | DOWNSTREAM |
| **chr18** | 47349684 | rs375530149 | C | G | *MYO5B* | NA | 4.500 | DOWNSTREAM |
| **chr18** | 47349933 | rs117972198 | G | A | *MYO5B* | NA | 4.000 | DOWNSTREAM |
| **chr18** | 47349776 | rs78201339 | A | G | *MYO5B* | NA | 3.000 | DOWNSTREAM |
| **chr18** | 47349653 | rs75335611 | C | T | *MYO5B* | NA | 2.333 | DOWNSTREAM |
| **chr18** | 47349945 | rs372605995 | T | A | *MYO5B* | NA | 2.333 | DOWNSTREAM |
| **chr18** | 47349683 | rs372278198 | C | T | *MYO5B* | NA | 2.000 | DOWNSTREAM |
| **chr18** | 47350945 | rs79745077 | G | T | *MYO5B* | NA | 0.800 | INTRON |
| **chr18** | 47813069 | rs200612776 | A | C | *CXXC1* | NA | 0.800 | DOWNSTREAM |
| **chr18** | 46196865 | rs74363574 | C | A | *RP11-426J5.2* | NA | 0.750 | DOWNSTREAM |
| **chr18** | 47350654 | rs375542187 | G | A | *MYO5B* | NA | 0.467 | UTR_3_PRIME |
| **chr18** | 43844715 | rs200571596 | G | T | *C18orf25* | NA | 0.000 | DOWNSTREAM |
| **chr18** | 47349692 | rs368309880 | T | G | *MYO5B* | NA | 0.000 | DOWNSTREAM |
| **chr18** | 47349739 | rs113215300 | G | T | *MYO5B* | NA | 0.000 | DOWNSTREAM |
| **chr18** | 47349768 | rs73959716 | A | C | *MYO5B* | NA | 0.000 | DOWNSTREAM |
| **chr18** | 47349841 | rs112057683 | G | A | *MYO5B* | NA | 0.000 | DOWNSTREAM |
| **chr18** | 47349867 | rs115831173 | T | C | *MYO5B* | NA | 0.000 | DOWNSTREAM |
| **chr18** | 47349881 | rs76811062 | A | G | *MYO5B* | NA | 0.000 | DOWNSTREAM |
| **chr18** | 47350658 | 0 | G | A | *MYO5B* | NA | 0.000 | DOWNSTREAM |
| **chr18** | 47350683 | rs74651470 | C | T | *MYO5B* | NA | 0.000 | DOWNSTREAM |
| **chr18** | 47350712 | rs73959721 | T | C | *MYO5B* | NA | 0.000 | DOWNSTREAM |
| **chr18** | 47350753 | rs75318758 | T | C | *MYO5B* | NA | 0.000 | DOWNSTREAM |
| **chr18** | 47350832 | rs79509391 | A | T | *MYO5B* | NA | 0.000 | DOWNSTREAM |
| **chr18** | 47350848 | rs75305404 | G | C | *MYO5B* | NA | 0.000 | DOWNSTREAM |
| **chr18** | 47350866 | rs75032645 | A | G | *MYO5B* | NA | 0.000 | DOWNSTREAM |
| **chr18** | 47350876 | rs78555149 | G | A | *MYO5B* | NA | 0.000 | DOWNSTREAM |
