# Supplementary figures and images for: Fine-Mapping of 18q21.1 Locus Identifies Single Nucleotide Polymorphisms Associated with Nonsyndromic Cleft Lip with or without Cleft Palate
Source: Front Genet. 2016 May 23;7:88. doi: 10.3389/fgene.2016.00088 (PMC4876112; doi:10.3389/fgene.2016.00088)

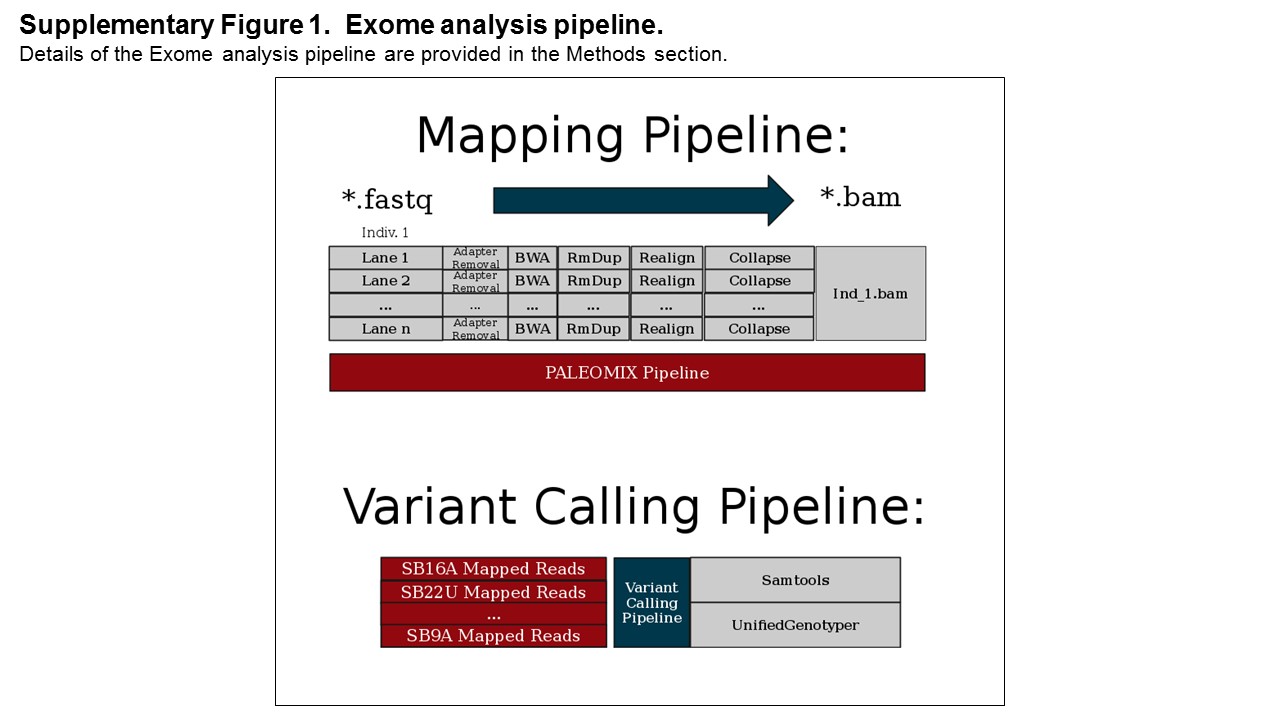

Supplement: Supplementary file 2 [file Image1.JPEG]
